# Validation of Ecological Momentary Assessment With Reference to Accelerometer Data: Repeated-Measures Panel Study With Multilevel Modeling

**DOI:** 10.2196/59878

**Published:** 2025-04-01

**Authors:** Jung Min Noh, SongHyun Im, JooYong Park, Jae Myung Kim, Miyoung Lee, Ji-Yeob Choi

**Affiliations:** 1 Department of Biomedical Sciences Seoul National University Seoul Republic of Korea; 2 Department of Big Data Medical Convergence Eulji University Seongnam Republic of Korea; 3 School of Exercise, Sport, and Health Sciences Oregon State University Corvallis, OR United States; 4 College of Physical Education and Sport Science Kookmin University Seoul Republic of Korea; 5 Cancer Research Institute Seoul National University Seoul Republic of Korea

**Keywords:** telemedicine, wearable electronic devices, physical activity, mobile phone, wearables, smartphones, ecological momentary assessment, EMA, global physical activity questionnaire, GPAQ, Bouchard’s physical activity, multilevel modeling, females, women, males, men, sensors, evaluation, comparative, South Korea

## Abstract

**Background:**

There is growing interest in the real-time assessment of physical activity (PA) and physiological variables. Acceleration, particularly those collected through wearable sensors, has been increasingly adopted as an objective measure of physical activity. However, sensor-based measures often pose challenges for large-scale studies due to their associated costs, inability to capture contextual information, and restricted user populations. Smartphone-delivered ecological momentary assessment (EMA) offers an unobtrusive and undemanding means to measure PA to address these limitations.

**Objective:**

This study aimed to evaluate the usability of EMA by comparing its measurement outcomes with 2 self-report assessments of PA: Global Physical Activity Questionnaire (GPAQ) and a modified version of Bouchard Physical Activity Record (BAR).

**Methods:**

A total of 235 participants (137 female, 98 male, and 94 repeated) participated in one or more 7-day studies. Waist-worn sensors provided by ActiGraph captured accelerometer data while participants completed 3 self-report measures of PA. The multilevel modeling method was used with EMA, GPAQ, and BAR as separate measures, with 6 subdomains of physiological activity (overall PA, overall excluding occupational, transport, exercise, occupational, and sedentary) to model accelerometer data. In addition, EMA and GPAQ were further compared with 6 domains of PA from the BAR as outcome measures.

**Results:**

Among the 3 self-reporting instruments, EMA and BAR exhibited better overall performance in modeling the accelerometer data compared to GPAQ (eg EMA daily: β=.387, *P*<.001; BAR daily: β=.394, *P*<.001; GPAQ: β=.281, *P*<.001, based on repeated-only participants with step counts from accelerometer as dependent variables).

**Conclusions:**

Multilevel modeling on 3 self-report assessments of PA indicates that smartphone-delivered EMA is a valid and efficient method for assessing PA.

## Introduction

A physical activity (PA) lifestyle has long been recognized as both a prerequisite for and predictor of maintaining good health. Decades of epidemiologic research have identified the preventive effects of PA against various physiological and mental health issues, including heart and other cardiovascular diseases [1-3], depression and suicidal thoughts [4-7], and cancer [2,8-10].

The recent development of digital and wearable technologies has made it possible to continuously track PA in real life through sensors embedded in digital devices. This expansion provides researchers with a broader range of choices, as both research-grade and consumer-grade wearables, with varying costs and capacities to measure health conditions, are now available in the market. While the potential benefits of these wearable technologies in research are substantial, it is essential to acknowledge several limitations: first, sensor-based measures pose challenges for large-scale epidemiological studies due to their associated costs and administrative difficulties in managing devices. Second, this approach is inadequate for capturing contextual information associated with the activity. Finally, there are limitations to the populations and circumstances capable of using health-tracking devices, creating potential risks for digital inequality [11-15]. Consequently, the advancement of sensor technologies does not diminish the importance of traditional report-based assessment methods for measuring PA.

The Global Physical Activity Questionnaire (GPAQ) is an instrument designed to collect self-reports on PA in the domains of occupational activity, travel, recreational activities (exercise), and sedentary behavior (SB) [16]. Comprising 16 questions, it is well-recognized for obtaining information on PA [17]. However, the retrospective approach adopted by the GPAQ entails an enhanced risk of memory bias and a lack of temporal specificity associated with the activity [18,19]. This limitation applies similarly to other measures that also rely on retrospective reports.

In contrast, the Bouchard [20] Physical Activity Record (BAR) offers a means for promptly gathering reports on PA. It allows respondents to record their PA at 15-minute intervals, rating their activity level on a scale from 1 (SB) to 9 (high-intensity exercise). However, the BAR is constrained by its reliance on traditional pen-and paper-recording methods and its log-based formats. As a result, respondents face relatively high demands in completing full sets of recordings, without the benefit of customized prompts when reporting their activities.

Smartphone-delivered ecological momentary assessment (EMA) addresses these limitations by providing a real-time and flexible assessment of ongoing PA. Although it may not directly serve as a substitute for international surveillance of PA, the potential of digitally delivered questionnaires lies in their ability to offer adaptive and diverse solutions compared with traditional methods, enabling the assessment of activities across a broader range of contexts. For example, previous studies have demonstrated the strengths of EMA over traditional reporting methods in assessing various clinical conditions across populations, including occupational stress in patients with cardiovascular disease [21], anxiety and depression [22-24], and general health-related quality of life [25]. The relative strengths of mobile-based questionnaires also include much easier storage, retrieval, and use of data over time.

However, there have been inconsistent findings regarding the agreement between momentary and recall-based assessment methods [26,27], and validation of the suggested approach for assessing PA remains limited. Addressing this gap is crucial to ensure that EMA methods are both reliable and robust, enabling researchers and practitioners to better understand PA patterns in real-world settings. By validating these methods against well-recognized self-report instruments such as the GPAQ and BAR, this study could support applied research across diverse clinical targets, offering improved guidelines for PA measurement. For these reasons, we aimed to validate EMA methods by comparing them with well-recognized self-report instruments for measuring PA: GPAQ and BAR. A multilevel analysis was conducted using the accelerometer and BAR as outcome variables to account for the hierarchical data structure inherent in the mixed-design study.

## Methods

### Recruitment

Participants were recruited between May and November 2015 from a public health center in Seoul, South Korea. Eligibility for study participation were as follows: adults (18 years of age or older) who voluntarily visited a center for nonorthopedic or neuromuscular causes, being capable of undertaking daily PA, having no plan for hospitalization during the study, and having access to their own mobile phones.

### Design and Procedures

**Figure 1:**
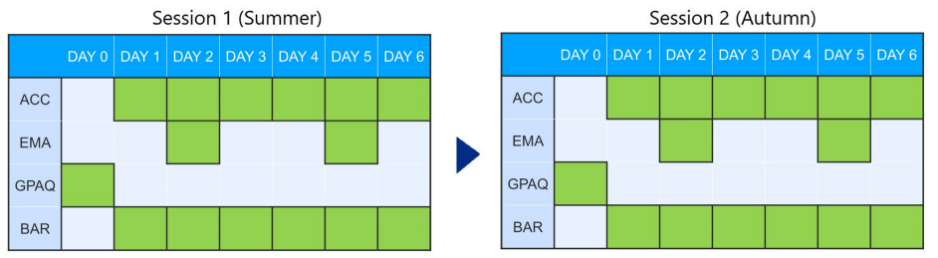
Design of a 7-day study. BAR: Bouchard’s physical activity record; EMA: ecological momentary assessment, GPAQ: global physical activity questionnaire.

A 7-day study was conducted during the May and November sessions of 2015. Participants were given the option to participate in one or both sessions. On day 0, participants’ body composition and handgrip strength were measured. Participants were asked to wear the ActiGraph GT3X+ (ActiGraph LLC) over the right waist within an anterior axillary line for 7 consecutive days (days 1-6). They were instructed to always wear the device while awake, except for water activities such as swimming and showering. Out of 3 self-report measures of PA (ie, EMA, GPAQ, and BAR) were completed according to the following timeline: EMA was completed on 1 weekday and 1 weekend day; GPAQ was completed on the first day before participants were provided with the waist sensor; BAR was completed daily between days 1 and 6 (Figure 1).

Days that participants provided data through each instrument are colored in grey. Accelerometer data were provided from days 1 to 6. Participants were asked to wear a waist sensor every day, except during swimming and showering. EMA was performed on 1 weekday and 1 weekend day. GPAQ was completed on day 0, and BAR data were collected between days 1 and 6. Participants who participated in at least one session are denoted as overall participants.

### Materials

#### The EMA

**Figure 2:**
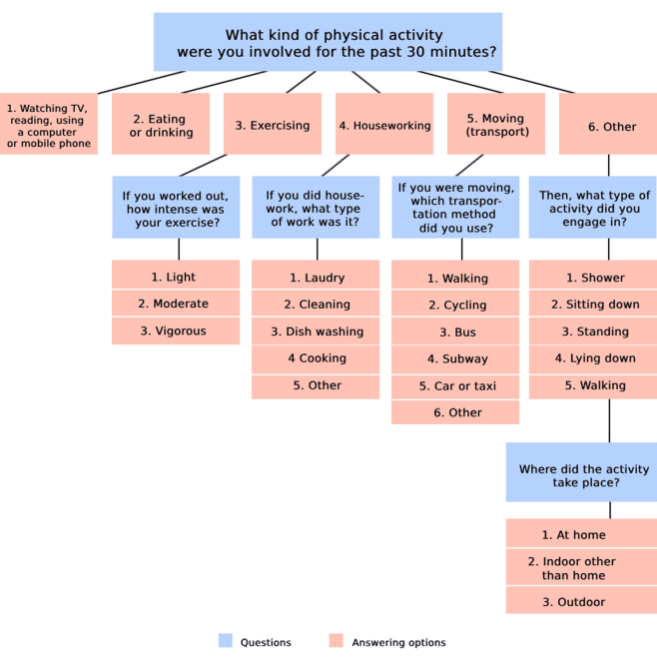
Flowchart of the EMA: ecological momentary assessment questionnaire.

EMA was generated through survey monkey. The assessment comprised 5 questions, with the specific questions displayed flexibly based on the respondent’s answer to the preceding question (Figure 2). PA questionnaires were sent to participants through SMS messages with a 2-hour interval between 8 AM and 10 PM on 1 weekday and 1 weekend. Upon receiving an alert, participants reported their primary PA during the preceding 30 minutes. Participants categorized their activities as either sedentary (sitting and lying down), transport, occupational (moderate or vigorous), or exercise (light, moderate, or vigorous). Total PA was calculated by summing these activity domains with the following coding scheme: sedentary=1; transport=4; moderate occupational=4; vigorous occupational=6; light and moderate exercise=4; vigorous exercise=6 [28].

#### The GPAQ

GPAQ [16,29] is a self-report assessment tool that collects data on 3 physical activity domains: occupational (moderate and vigorous), recreational (moderate and vigorous), and transport, as well as the information on SB. The questionnaire consists of 16 questions that examine the amount of time (in minutes) spent on each activity domain during a typical week. To calculate the overall metabolic equivalents of tasks (METs), the time spent on moderate and transportation PA was multiplied by 4 METs, while the time spent on vigorous PA was multiplied by 8 METs. Sedentary time was assessed by asking about the duration spent sitting or reclining on a typical day. To estimate the average daily time spent on moderate and vigorous PA, the time spent on those intensity PA was divided by 7. The Korean version of GPAQ, previously validated for native Korean respondents [29], was used in this study.

#### The BAR

BAR [20] is a commonly used self-reporting method where participants record their PA for each 15-minute interval over a span of 3 days. Activities are rated on a scale from 1 to 9 (1 indicating sedentary activity and 9 indicating intense work or high-intensity exercise) to generate a total PA score. In this study, the questionnaire items in BAR [20] were adapted to gather participants’ estimates of total moderate-to-vigorous PA (MVPA), moderate and vigorous occupational activity, transport (movement), moderate and vigorous exercise (leisure-related activity), and SB. Participants, encouraged to report their activity every hour, were asked to complete BAR [20] to record their PA over 24 hours for 6 consecutive days.

#### Accelerometer

Accelerometer data was collected using ActiGraph GT3X+, a triaxial accelerometer which is a valid method to assess daily free-living PA [30]. The device is characterized by its compact size (4.6 cm × 3.3 cm × 1.5 cm) and lightweight design (19 g). The accelerometer collects the acceleration signal at a 30 Hz sampling rate. The acceleration signal is summed over a 60-second time interval (epoch) and stored as activity counts. In this study, we used the estimation of MVPA time, sedentary time, number of steps, and METs. The PA intensity was classified using a Freedson [31] algorithm, MVPA: >2689 counts per minute (CPM); the sedentary time was defined as <100 CPM (Troiano 2008). The METs variable was estimated using a Freedson [31] equation, 1.439008 + (0.000795× counts/min).

### Statistical Analysis

**Figure 3:**
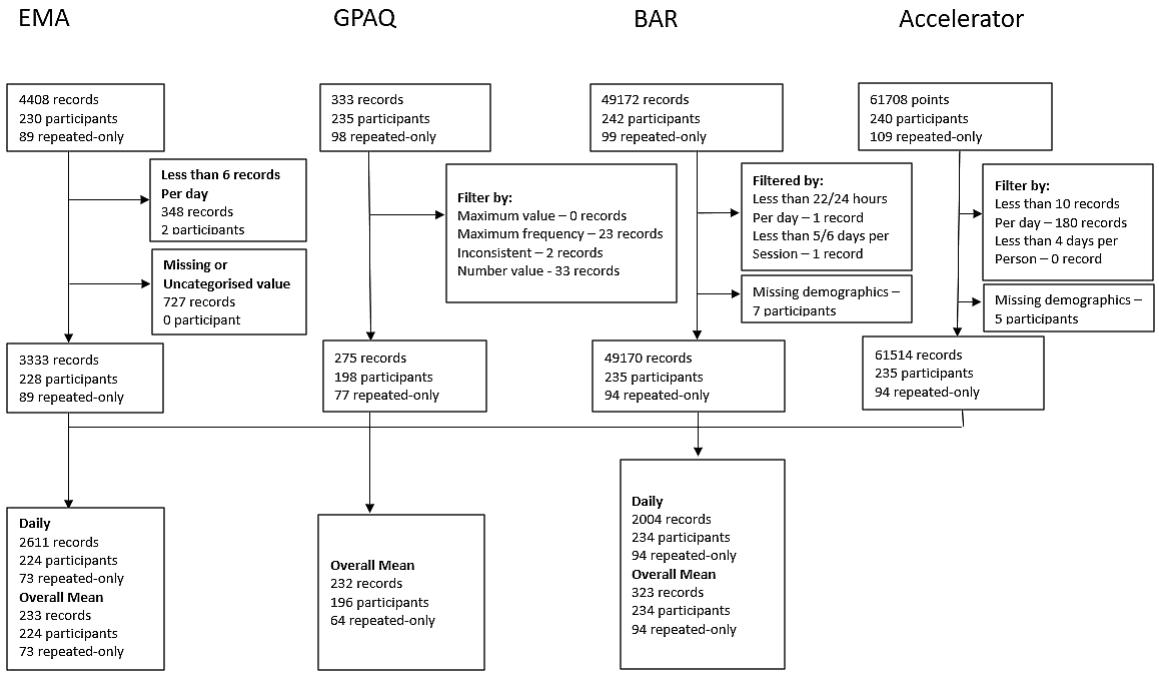
Flow chart of data cleaning and integration for the multilevel analysis with the accelerometer data as outcome measures. BAR: Bouchard’s physical activity record; EMA: ecological momentary assessment, GPAQ: global physical activity questionnaire.

The data cleaning process is illustrated in Figure 3. Of the 4408 EMA records (n=230), 348 records (n=2) were eliminated as participants provided less than 6 reports per day (ie, 6/8, less than 75%). In addition, 727 records were removed due to missing or uncategorized values, resulting in 3333 records (n=228). On the GPAQ, of the 333 records (n=235), 58 records were excluded based on the GPAQ analysis guideline [16], leaving 275 records (n=198).

To address repeated measures within participants, the multilevel analysis method was used with (1) step counts and total MVPA minutes from the accelerometer data and (2) activity-specific domains from the BAR as outcome measures. Multilevel modeling is suitable for addressing the hierarchical data structure inherent in the mixed-design study, which includes both within- and between-participant measures [31].

On the analysis with accelerometer data as outcome measures, both the BAR and EMA data were structured into 2 levels. Daily observations formed the lowest level of the hierarchy (level 1), nested within individuals (level 2). Data driven from 3 self-report instruments (EMA, GPAQ, and BAR), each with 6 PA domains (total MVPA; total MVPA excluding occupational; transport; leisure time exercise; occupational PA; and SB), were separately used to assess the association with objectively obtained accelerometer data (steps counts and total MVPA minutes). Both EMA and the BAR data were analyzed in 2 variations based on time intervals: daily and overall mean scores per participant. Filtered EMA, GPAQ, and BAR data were separately integrated with accelerometer data based on matching dates. The final analysis included 3 datasets each containing 2593 (EMA-accelerometer: n=218, 89 repeated), 270 (GPAQ-accelerometer: n=196, 77 repeated), and 2004 (BAR-accelerometer: n=234, 94 repeated) records.

Compared to the EMA method, where participants provide data at 2-hour intervals between 8 AM and 10 PM, the BAR captures PA continuously throughout the day, with reports recorded every 15 minutes. We further performed multilevel modeling using activity-specific domains from the BAR data as outcome measures to evaluate the validity of EMA against the more fine-grained reporting intervals of the BAR. Unlike accelerometer data, which measures PA only through step counts or total MVPA minutes, the BAR offers an additional advantage by allowing for domain-specific validation. For example, SB reported through EMA can be compared with SB reported through BAR. Similar time-slot matching methods were applied to integrate the BAR data with both EMA and GPAQ datasets. The final dataset sizes were 539 (EMA-BAR: n=221, with 73 repeated measures) and 198 (GPAQ-BAR: n=198, with 74 repeated measures).

Standardized coefficients were calculated for the comparisons between different instruments. Participants’ age and gender were included as covariates to account for differences in outcomes attributed to demographics. A sensitivity analysis on EMA data indicated that there is the difference in the validity of EMA data based on reported days, with reports from weekdays showing better match with outcome measures compared with those from weekend days. Therefore, for the analysis of EMA data, the type of the day (week vs weekend day) was further used as a covariate alongside participants’ age and gender. Total and repeated-only participant (those who enrolled in both May and November sessions of the study) groups were separately examined.

### Ethical Considerations

This study adhered to ethical guidelines for research involving human participants and received approval from the institutional review board at Seoul National University Hospital, Seoul, Korea 1812-129-997). All methods were carried out in accordance with the guidelines proposed in the Declarations of Helsinki. A total of 241 participants provided informed consent.

## Results

### Participant Characteristics

#### Overview

A total of 235 participants (137 female, 98 male, and 94 repeated) participated in the study. The mean age of participants was 52.71 (SD 9.07). The characteristics of these participants are shown in Table 1. The majority of participants were married (200/235, 85.11%) at the time of the study and had no smoking experience (170/235, 72.34%). All participants completed at least secondary level education and a larger proportion of participants (117/235, 49.79%) completed a higher-level education. Of the participants who enrolled in both May and November sessions of the study (94; denoted as repeated-only participants), the mean age was 52.10 (SD 7.50) years. The majority of participants were married (85/94, 90.43%) females (80/94, 85.11%), who completed a higher-level education (55/94, 58.51%) and had no smoking experience (81/94, 86.17%).

**Table 1:** Characteristics of participants enrolled in the study (May-July and Sept-Nov 2015).

Characteristics		Total (N=235)	Repeated-only (n=94)
**Sex, n (%)**			
	Female	161 (68.51)	80 (85.11)
	Male	74 (31.49)	14 (14.89)
**Age (years), n (%)**			
	<50	86 (36.60)	39 (41.49)
	50-59	98 (41.70)	34 (36.17)
	More than 60	51 (21.70)	21 (22.34)
**Education, n (%)**			
	<Middle school	24 (10.21)	4 (4.26)
	High school	93 (39.57)	34 (36.17)
	More than college	117 (49.79)	55 (58.51)
	Missing	1 (0.43)	1 (1.06)
**Marriage status, n (%)**			
	Unmarried	18 (7.66)	4 (4.26)
	Married	200 (85.11)	85 (90.43)
	Others	17 (7.23)	5 (5.32)
**Monthly income (KRW; 1 KRW=US $0.00069), n (%)**			
	<200	38 (16.17)	18 (19.15)
	200-400	117 (49.79)	41 (43.62)
	More than 400	77 (32.77)	34 (36.17)
	Missing	3 (1.28)	1 (1.06)
**Job status, n (%)**			
	Office	95 (40.43)	33 (35.11)
	Manual	64 (27.23)	19 (20.21)
	Housewives	54 (22.98)	30 (31.91)
	Others	21 (8.94)	12 (12.77)
	Missing	1 (0.42)	—
**Smoking, n (%)**			
	Never	170 (72.34)	81 (86.17)
	Ex-smoker	33 (14.04)	10 (10.64)
	Current	27 (11.49)	1 (1.06)
	Missing	5 (2.13)	2 (2.13)
**Alcohol, n (%)**			
	Never	93 (39.57)	34 (36.17)
	Ex-drinker	18 (7.66)	5 (5.32)
	Current	119 (50.64)	53 (56.38)
	Missing	5 (2.13)	2 (2.13)
**BMI, n (%)**			
	<23	96 (40.85)	39 (41.49)
	23-25	61 (25.96)	25 (26.60)
	More than 25	66 (28.09)	22 (23.40)
	Missing	12 (5.11)	8 (8.51)

Participants who participated in at least one session are denoted as total participants; participants who participated in both sessions are denoted as repeated-only participants.

#### Average Time Engaged in Various Physical Activity Types

The mean durations of time (minutes per day) spent in each activity type, measured through different instruments (EMA, GPAQ, BAR, and accelerometer), are summarized in Tables 2 and 3. A rank-based comparison of self-report instruments indicated that the time spent on SB took the largest proportion within each instrument. Similarly, leisure exercise took the smallest proportion across different self-report measures of PA On EMA and BAR, transport time were reported over the time spent on occupational activities, while occupational activities were reported over transport time on GPAQ. Across all self-report methods, participants underreported the time spent on SB (Mean_EMA_ 251.31, SD_EMA_ 68.53; Mean_GPAQ_ 457.27, SD_GPAQ_ 257.28; Mean_BAR_ 502.0, SD_BAR_ 141.58), compared with the SB tracked through wearable sensors (Mean 1048.02, SD 208.77), while overreporting the time spent on total MVPA (Mean_accelerometer_ 104.38, SD_accelerometer_ 102.38 versus Mean_EMA_ 194.41, SD_EMA_ 68.14; Mean_GPAQ_ 146.17, SD_GPAQ_ 155.21; Mean_BAR_ 146.67, SD_BAR_ 91.16).

**Table 2:** Duration (minutes per day) of engaging in different physical activity types by accelerometer and EMA^a^.

	Accelerometer			EMA (daily)			EMA (overall mean)	
	Total (N=235), mean (SD)	Repeat (N=94), mean (SD)	Total (N=224), mean (SD)		Repeat (N=73), mean (SD)	Total (N=244), mean (SD)		Repeat (N=73), mean (SD)
Total	104.38 (102.38)	99.93 (91.41)	195.19 (107.64)		203.94 (107.47)	194.41 (68.14)		202.25 (69.90)
MET^b^	25.79 (3.87)	25.58 (3.61)	N/A^c^		N/A	N/A		N/A
Steps	8385.32 (5195.79)	8520.92 (5255.28)	N/A		N/A	N/A		N/A
Occu^d^	N/A	N/A	72.92 (80.44)		87.74 (84.30)	67.79 (57.17)		86.78 (54.90)
Trans^e^	N/A	N/A	93.15 (91.28)		92.80 (91.48)	94.97 (57.95)		93.40 (48.10)
Exer^f^	N/A	N/A	29.12 (53.04)		23.39 (49.56)	31.66 (40.29)		22.07 (26.43)
SB^g^	1048.02 (208.77)	1033.39 (205.46)	251.02 (106.85)		245.53 (107.09)	251.31 (68.53)		247.21 (70.96)

^a^EMA: ecological momentary assessment.

^b^MET: metabolic equivalent of task.

^c^N/A: not available.

^d^Occu: occupational physical activity.

^e^Trans: transport physical activity.

^f^Exer: leisure-time exercise.

^g^SB: sedentary behavior.

**Table 3:** Duration (minutes per day) of engaging in different physical activity types by the GPAQ^a^ and BAR^b^.

	GPAQ		BAR (daily)		BAR (overall mean)	
	Total (N=198), mean (SD)	Repeat (N=77), mean (SD)	Total (N=238), mean (SD)	Repeat (N=94), mean (SD)	Total (N=238), mean (SD)	Repeat (N=94), mean (SD)
Total	146.17 (155.21)	124.89 (97.70)	150.0 (130.23)	155.29 (124.42)	146.67 (91.16)	155.76 (78.45)
MET^c^	N/A^d^	N/A	N/A	N/A	N/A	N/A
steps	N/A	N/A	N/A	N/A	N/A	N/A
Occu^e^	62.92 (115.05)	52.67 (73.52)	56.27 (98.59)	61.11 (87.34)	53.75 (68.49)	61.3 (50.14)
Trans^f^	37.84 (52.74)	42.52 (44.23)	65.31 (73.98)	67.82 (77.96)	64.44 (47.75)	68.06 (43.11)
Exer^g^	32.54 (51.06)	31.36 (49.78)	28.41 (58.1)	26.36 (55.13)	28.48 (41.35)	26.4 (35.31)
SB^h^	457.27 (257.28)	477.27 (259.21)	504.69 (190.63)	511.99 (183.17)	502.0 (141.58)	511.26 (121.03)

^a^GPAQ: global physical activity questionnaire.

^b^BAR: Bouchard’s physical activity record.

^c^MET: metabolic equivalent of task.

^d^N/A: not available.

^e^Occu: occupational physical activity.

^f^Trans: transport physical activity.

^g^Exer: leisure-time exercise.

^h^SB: sedentary behavior.

### Multilevel Modeling With Accelerometer Data as Outcome Measures

#### Overview

Results of multilevel modeling with the measurement outcomes from 3 self-report instruments (EMA, GPAQ, and BAR) are shown in Tables 4 and 5. In general, EMA and BAR exhibited better overall performance in modeling the accelerometer data compared with GPAQ (eg, EMA daily: β=.387, *P*<.001; BAR daily: β=.394, *P*<.001; GPAQ: β=.281, *P*<.001, based on repeated-only participants with steps counts from the accelerometer as dependent variables; Table 4). Similar results were found with a total MVPA as dependent measures (eg, EMA daily: *β*=.367, *P*<.001; BAR daily: β=.358, *P*<.001; GPAQ: β=.280, *P*<.001; based on repeated-only participants; Table 5).

**Table 4:** Main outcomes of multilevel modeling with the accelerometer data (steps counts) as dependent measures.

		EMA^a^ (daily)		EMA (overall mean)		GPAQ^b^		BAR^c^ (daily)		BAR (overall mean)	
		*β*	*P* value	β	*P* value	β	*P* value	β	*P* value	β	*P* value
**Total participants**											
	Total MVPA^d^	.245	.001	.145	.03	.087	.001	.323	.001	.291	.001
	Total exc occu^e^	.197	.001	.062	.36	.105	.001	.422	.001	.380	.001
	Occu^f^	–.031	.48	–.061	.38	.042	.03	.035	.111	.048	.34
	Trans^g^	.185	.001	.073	.28	.104	.001	.312	.001	.268	.001
	Exer^h^	.230	.001	.132	.05	.101	.001	.282	.001	.306	.001
	Sitting/lying^i^	–.164	.001	–.090	.17	–.111	.001	–.112	.001	–.148	.004
**Repeated-only participants**											
	Total MVPA	.387	.001	.287	.02	.281	.001	.394	.001	.335	.001
	Total exc occu	.297	.001	.154	.19	.280	.001	.484	.001	.408	.001
	Occu	–.020	.75	.384	.77	.084	.28	.028	.35	.032	.65
	Trans	.212	.001	.475	.68	.227	.002	.370	.001	.312	.001
	Exer	.348	.001	.311	.01	.183	.02	.296	.001	.287	.001
	Sitting and lying	–.202	.001	–.186	.11	–.157	.04	–.194	.001	–.236	.001

^a^EMA: ecological momentary assessment.

^b^GPAQ: global physical activity questionnaire.

^c^BAR: Bouchard’s physical activity record.

^d^Total MVPA: total moderate-to-vigorous physical activity.

^e^Total exc occu: total physical activity excluding occupational.

^f^Occu: occupational physical activity.

^g^Trans: transport physical activity.

^h^Exer: leisure-time exercise.

^i^Sitting or lying: sitting or lying down (sedentary behavior).

**Table 5:** Main outcomes of multilevel modeling with the accelerometer data (total MVPA^a^) as dependent measures.

		EMA^b^ (daily)			EMA (overall mean)			GPAQ^c^			BAR^d^ (daily)			BAR (overall mean)	
		Β	*P* value	β		*P* value	β		*P* value	β		*P* value	β		*P* value
**Total participants**															
	Total MVPA	.220	.001	.088		.19	.079		.001	.259		.001	.239		.001
	Total exc occu^e^	.113	.006	–.023		.74	.129		.001	.387		.001	.352		.001
	Occu^f^	–.029	.50	–.145		.04	.007		.73	–.017		.44	.005		.91
	Trans^g^	.084	.04	-.014		.84	.068		.001	.253		.001	.249		.001
	Exer^h^	.252	.001	.206		.002	.134		.001	.308		.001	.286		.001
	Sitting or lying^i^	–.060	.14	–.003		.97	–.058		.004	–.014		.53	–.051		.30
**Repeated-only participants**															
	Total MVPA	.367	.001	.199		.10	.280		.001	.358		.001	.316		.001
	Total exc occu	.275	.001	.053		.66	.319		.001	.483		.001	.446		.001
	Occu	.022	.73	–.012		.93	.064		.41	–.020		.50	–.027		.69
	Trans	.111	.06	–.058		.62	.181		.01	.319		.001	.336		.001
	Exer	.363	.001	.359		.001	.277		.001	.370		.001	.330		.001
	Sitting or lying	–.112	.06	–.092		.43	–.126		.09	–.149		.001	–.198		.002

^a^Total MVPA: total moderate-to-vigorous physical activity.

^b^EMA: ecological momentary assessment.

^c^GPAQ: global physical activity questionnaire.

^d^BAR: Bouchard’s physical activity record.

^e^Total exc occu: total physical activity excluding occupational.

^f^Occu: occupational physical activity.

^g^Trans: transport physical activity.

^h^Exer: leisure-time exercise.

^i^Sitting or lying: sitting or lying down (sedentary behavior).

On both EMA and BAR, the daily-based measures exhibited better performance than the overall mean per participants (eg, EMA daily: β=.245, *P*<.001 vs EMA mean: β=.145, *P*=.03; BAR daily: β=.323, *P*<.001 vs BAR mean: β=.291, *P*<.001; based on total participants with steps counts from accelerometer as dependent variables; Table 4). Among the total and repeated-only participant groups, data driven from the repeated-only participant group usually showed higher performance than the data from the total participant group (eg, EMA daily total: β=.220, *P*<.001 vs EMA repeated-only: β=.367, *P*<.001, based on total MVPA from accelerometer as dependent variables; Table 5). Among different domains of PA, occupational PA generally showed lower performance in modeling the accelerometer data compared with transport and leisure-time exercise (eg, EMA daily occupational PA: β=.022, *P*=.732 vs EMA transport PA: β=.111, *P*=.06, EMA leisure time exercise: β=.363, *P*<.001, based on repeated-only participants with total MVPA from accelerometer as dependent variables; Table 5).

#### Multilevel Modeling With the BAR as Outcome Measures

Results of multilevel modeling with activity-specific domains from the BAR as outcome measures are shown in Table 6. On overall participant group, EMA exhibited better overall performance in modeling outcome measures compared to the GPAQ (EMA daily: β=.311, *P*<.001; GPAQ mean: β=.294, *P*<.001, based on total participants; Table 6). By contrast, results with repeated-only participants showed better performance of the GPAQ (β=.495, *P*<.001) over all 3 variations of EMA (EMA daily: β=.436, *P*<.001, mean: β=.349, *P*<.001). Similar to the results of the analysis with the accelerometer as an outcome measure, general performance was better with repeated-only participants compared to the participant group that includes both of those who participated once or twice in the study.

**Table 6:** Main outcomes of multilevel modeling with the BAR^a^ data as dependent measures.

		EMA^b^ (daily)		EMA (overall mean)		GPAQ^c^		
		β	*P* value	β	*P* value		β	*P* value
**Total participants**								
	Total MVPA^d^	.311	.001	.202	.001		.284	.001
	Total exc occu^e^	.139	.001	.135	.001		.280	.001
	Occu^f^	.188	.001	.264	.001		.296	.001
	Trans^g^	.215	.001	.094	.19		.089	.28
	Exer^h^	.311	.001	.272	.001		.490	.001
	Sitting or lying^i^	.177	.001	.130	.05		.331	.001
**Repeated-only participants**								
	Total MVPA	.436	.001	.349	.001		.495	.001
	Total exc occu	.211	.001	.300	.02		.376	.004
	Occu	.174	.004	.357	.003		.558	.001
	Trans	.265	.001	.134	.31		.233	.08
	Exer	.403	.001	.510	.001		.693	.001
	Sitting or lying	.268	.001	.315	.007		.382	.001

^a^BAR: Bouchard’s physical activity record.

^b^EMA: ecological momentary assessment.

^c^GPAQ: global physical activity questionnaire.

^d^Total MVPA: total moderate-to-vigorous physical activity.

^e^Total exc occu: total physical activity excluding occupational.

^f^Occu: occupational physical activity.

^g^Trans: transport physical activity.

^h^Exer: leisure-time exercise.

^i^Sitting or lying: sitting or lying down (sedentary behavior).

## Discussion

### Principal Findings

We conducted a comprehensive comparison of different assessment instruments (EMA, GPAQ, BAR, and accelerometer) to validate the EMA methodology for assessing PA. Compared to international surveillance methods for assessing PA, the smartphone-delivered EMA method demonstrated strengths across various PA metrics. This was evident both through analysis using objectively measured accelerometer data as reference measures and through analysis using well-validated, log-like recording systems as reference measures.

### Interpretation of Findings

We observed consistent patterns in participants’ daily activity reports and their associations with accelerometer data collected via waist sensors. Generally, participants tended to overestimate total MVPA minutes spent compared to those tracked by wearable sensors. This aligns with previous research findings that reported overestimation of activity levels through self-reported measures over sensor-derived outcomes [32-34].

Comparisons among the 3 self-report instruments for modelling accelerometer data suggested that assessments with smaller time intervals yield stronger outcomes. The superior performance of the BAR and EMA over the GPAQ demonstrates the benefits of higher reporting frequencies and shorter recall periods. The results also indicate that daily-based measures on both BAR and EMA are more effective than the overall mean per participant, underscoring the appropriateness of multilevel modeling approaches. These findings have significant implications for research design, suggesting that instruments using shorter time intervals and daily reporting protocols are more suitable for capturing nuanced variations in PA patterns.

The practical advantages of EMA are particularly noteworthy in this context. In the current study, EMA allows up to 7 recordings per day, each covering the past 30 minutes, offering a compromise between accuracy and feasibility compared with the more demanding BAR, which requires participants to record data every 15 minutes. This balance makes EMA especially valuable in large-scale health research or clinical applications, where participant burden is a critical consideration. For instance, EMA’s ability to deliver reliable data with reduced respondent demands could improve adherence rates in long-term studies, such as those monitoring PA in individuals with chronic conditions like diabetes or cardiovascular disease. In clinical practice, this approach could help health care providers better tailor interventions by obtaining more precise and contextually relevant activity data.

Moreover, the flexibility of EMA offers opportunities to enhance the contextual understanding of PA. Unlike the BAR and GPAQ, EMA can capture additional contextual information, such as location, social settings, or emotional states during PA. This capability has practical applications in designing personalized behavioral interventions. For example, EMA prompts could be used to deliver real-time feedback or motivational messages when a participant is detected to be sedentary or transitioning to PA, leveraging data from wearable devices synced with smartphones. This adaptability positions EMA as a promising tool for both preventive health strategies and rehabilitation programs aimed at improving PA behaviors.

This study demonstrated a relatively high data completion rate, with most participants providing full sets of data across the different measuring instruments (Figure 3). However, future studies should focus on optimizing the temporal intervals of PA measurement to identify a balance between measurement accuracy and participant burden [35]. For example, investigating whether hourly prompts yield comparable validity to those at 30-minute intervals could inform the design of more efficient EMA protocols. Such refinements could enhance EMA’s utility across varied populations and contexts, including both high-demand clinical environments and large-scale epidemiological studies.

### Limitations

The recruitment for this study was conducted at a single public health center, where participants volunteered to take part in the study. While using this recruitment strategy was necessary to validate the Physical Activity Questionnaire [36] used in previous studies, a significant proportion of the participants were married females aged 50 years or older, indicating a potential skew in the overall participant demographic. This suggests that the tendencies observed in participants’ engagement in different types of activity, as well as the relatively low performance of occupational activity in modeling outcomes measures compared with other PA domains, may not be fully generalizable [32,37]. In addition, due to technological limitations, we did not prompt participants to report their PA through EMA at random intervals throughout the day. As a result, there is a possibility that the data may be skewed toward periods of more representative PA. Future studies should consider adopting methodologies that allow for more flexible timing of EMA prompts to address this limitation.

### Conclusions

Overall, our comprehensive comparison of different assessment instruments underscores smartphone-delivered EMA as a valid and consistent method for assessing PA across various domains. While there is growing interest in using accelerometers for passive PA assessment, challenges associated with sensor-based measures highlight the continued need for self-report instruments in PA measurement. Smartphone-delivered EMA offers an additional advantage over traditional self-report tools by enabling real-time, adaptive monitoring of PA, while also allowing for more efficient data storage, retrieval, and use. In summary, the robustness of EMA in assessing various PA domains, coupled with its potential to collect contextual information, positions EMA as a valid and effective tool for the assessment of PA.

## Abbreviations

BAR: Bouchard’s physical activity record
CPM: counts per minute
EMA: ecological momentary assessment
GPAQ: Global Physical Activity Questionnaire
MET: metabolic equivalent of task
MVPA: moderate-to-vigorous physical activity
PA: physical activity
SB: sedentary behavior

## Data Availability

The datasets generated and/or analyzed during the current study are available upon request to the corresponding author.
